# Long-Term Leaching Behavior of Organic and Inorganic Pollutants after Wet Processing of Solid Waste Materials

**DOI:** 10.3390/ma15030858

**Published:** 2022-01-23

**Authors:** Maria Prieto-Espinoza, Bernd Susset, Peter Grathwohl

**Affiliations:** 1 Center for Applied Geosciences, University of Tübingen, Schnarrenbergstr. 94-96, 72076 Tübingen, Germany; prietoespinoza@unistra.fr (M.P.-E.); bernd.susset@ifg.uni-tuebingen.de (B.S.); 2 Institute Earth and Environment (ITES UMR 7063), University of Strasbourg, CNRS/EOST, 5 Rue Descartes, 67084 Strasbourg, France

**Keywords:** mineral recycling material, leaching test, heterogeneity, compliance testing

## Abstract

The recycling of mineral materials is a sustainable and economical approach for reducing solid waste and saving primary resources. However, their reuse may pose potential risks of groundwater contamination, which may result from the leaching of organic and inorganic substances into water that percolates the solid waste. In this study, column leaching tests were used to investigate the short- and long-term leaching behavior of “salts”, “metals”, and organic pollutants such as PAHs and herbicides from different grain size fractions of construction & demolition waste (CDW) and railway ballast (RB) after a novel treatment process. Specifically, silt, sand and gravel fractions obtained after a sequential crushing, sieving, and washing process (“wet-processing”) of very heterogeneous input materials are compared with respect to residual contamination, potentially limiting their recycling. Concentrations in solid fractions and aqueous leachate were evaluated according to threshold values for groundwater protection to identify relevant substances and to classify materials obtained for recycling purposes according to limit values. For that, the upcoming German recycling degree was applied for the first time. Very good agreement was observed between short and extensive column tests, demonstrating that concentrations at L/S 2 ratios are suitable for quality control of recycling materials. Different solutes showed a characteristic leaching behavior such as the rapid decrease in “salts”, e.g., SO_4_^2−^ and Cl^−^, from all solid fractions, and a slower decrease in metals and PAHs in the sand and silt fractions. Only the gravel fraction, however, showed concentrations of potential pollutants low enough for an unlimited re-use as recycling material in open technical applications. Sand fractions may only be re-used as recycling material in isolated or semi-isolated scenarios. Leaching from heterogeneous input materials proved harder to predict for all compounds. Overall, column leaching tests proved useful for (i) initial characterization of the mineral recycling materials, and (ii) continuous internal (factory control) and external quality control within the upcoming German recycling decree. Results from such studies may be used to optimize the treatment of mixed solid waste since they provide rapid insight in residual pollution of material fractions and their leaching behavior.

## 1. Introduction

The largest solid waste stream in Germany with an annual volume of more than 275 million tones comprises 32% of construction and demolition waste (CDW), of which about 90% is reused [1]. Recycling mineral waste has a lot of advantages in terms of sustainability and economical aspects. To increase recycling potential, more and more companies start to treat excavated soil-stone mixtures, and demolition waste or railway ballast, combining crushing with dry and wet sieving and washing processes (wet processing). However, the reuse of mineral materials may pose potential risks of environmental pollution, resulting from leaching of organic and inorganic substances into percolating water and, ultimately, into groundwater [2,3,4,5]. 

In this context, the risk concerning potential contaminants in such materials must be addressed by their leaching potential into water rather than assessing total concentrations in the solid phase. Column leaching tests and batch shaking tests are frequently used to assess the transfer of contaminants into water [3,6,7]. Column leaching tests are preferred because they allow for assessing time-dependent behavior and simulating the flow of water through solid materials closer to natural conditions [8,9]. In Germany, the standard DIN 19528 (2009) is used for examining the leaching potential of inorganic and organic substances from solid materials [10]. Generally, “extensive column tests” are performed to characterize and evaluate the long-term leaching behavior of contaminants in which eluates are collected at different liquid-to-solid (L/S) ratios (e.g., 0.3, 1, 2, 4 and 10 L/kg). L/S ratios represent the time after which a certain volume of water has percolated through the solid material in the column (in L/kg dry matter). For compliance testing, “short column tests” may be employed that provide results of cumulative concentrations at one fixed L/S of 2 L/kg, which then are compared with threshold values set by special regulations [5,9,11]. Initially, equilibrium concentrations are often observed in column eluates [12]. The decrease in concentrations with increasing L/S ratios (or time) may be due to depletion of highly soluble substances, or a shift to non-equilibrium conditions because of mass transfer limitations (e.g., slow intraparticle diffusion) indicated by an extended tailing of the solute concentrations in the leachate [13]. The shift between equilibrium to non-equilibrium conditions may depend on initial conditions [12], flow velocities, grain-sizes, sorption capacity and contaminant release kinetics [8,9,12]. Typically, three basic leaching scenarios can be described for (i) fast leaching substances such as “salts” (e.g., sodium, potassium, chloride), where a rapid decline in concentrations in column effluents is observed (at L/S < 2 L/kg); (ii) intermediate compounds such as some metals, where mass release is governed by leaching parameters such as pH, redox conditions, ionic strength and DOC-complexations [14,15,16,17], and; (iii) for strongly sorbing compounds such as PAHs, where equilibrium concentrations prevail over extended periods of time [3]. 

Column leaching tests are thereby proposed as a common procedure for the evaluation of environmental qualities of solid waste recycling materials [5,9,18]. In Germany, the upcoming recycling directive [19] is based on improved methods for groundwater risk assessment to derive a new regulatory framework for the reuse of solid waste materials. For a given substance, the concentration level avoiding any significant alteration of the chemical status of groundwater is defined as the “insignificance threshold” concentration (“GFS”, in German “Geringfügigkeitsschwelle”) [20]. The GFS values are based on eco- and human toxicological tests and are not intended to set a quality goal for groundwater, but rather reflect a groundwater status unaffected by human activity [20]. 

Concentration limits at L/S 2 eluates are set depending on the type of mineral recycling material (e.g., CDW, RB, steel slag etc.), the type of technical application (open technical applications and isolated or semi-isolated technical applications protected from seepage water), the distance to the groundwater table, and the soil characteristics of the underground [19]. If the quality of the recycling material shows high variability, different “material classes” are defined by different sets of limit concentrations in eluates from the same mineral recycling material, so-called “material values” (e.g., material class RC–1, highest quality) [11]. The comparison of concentrations in L/S 2 eluates with GFS values and/or limit values of “material classes” will ultimately define permissible applications of the mineral recycling material. This concept is implemented in Germany within the upcoming recycling directive [19] with a quality assurance system and material-specific testing programs, where the quality of mineral recycling materials is assessed based on extensive column percolation tests [10]. Furthermore, short-term column percolation tests [10] are performed for internal and continuous external quality control [11].

In this study, short and extensive column tests were performed to examine the leaching of organic and inorganic substances from railway ballast (RB), and construction and demolition waste (CDW), which both underwent a sophisticated washing and grain size separation process, so-called wet processing. The input material (‘In’, highly heterogeneous RB or CDW) as well as its separated grain-size fractions such as silt (‘U’, <0.063 mm), sand (‘S’, 0–2 mm) and gravel (‘G’, 2–8 mm) were examined. The aim of this study was (i) to characterize comprehensively the leaching behavior of organic and inorganic contaminants from input and recycled material fractions (i.e., RB and CDW); (ii) to assess the quality of the different grain size fractions with respect to threshold values for potential risks of groundwater contamination and material values; (iii) to compare results of the short and extensive tests, and; (iv) to examine the long-term leaching behavior of the investigated substances.

## 2. Materials and Methods

### 2.1. Materials

**Figure 1:**
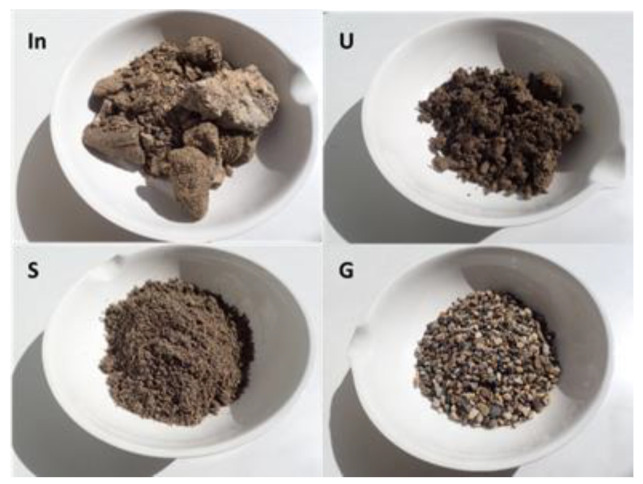
Solid waste fractions of construction and demolition waste (CDW3) as received from the recycling plant: input material (In) as well as silt (U), sand (S) and gravel (G) fractions obtained after wet-treatment.

In total, 3 sets of construction and demolition waste (CDW1, CDW2 and CDW3) and 1 set of railway ballast (RB) were examined to reflect different sources of recycling materials and their variability. Samples were collected in December 2017 (RB and CDW1), March 2018 (CDW2) and May 2018 (CDW3). The original CDW material was a mixture of soil, demolition and construction waste. At the recycling plant, input materials were crushed and “cleaned” in a complex washing process. All solid waste materials were separated into different grain size fractions (Figure 1). Large fragments were sieved into different gravel-size fractions: 32–50 mm, 16–32 mm, 8–16 mm and 2–8 mm. Smaller particles in suspension underwent a centrifugation process, wherein the sand fraction (0–2 mm) was separated and further washed. Finally, the silt fraction (<0.063 mm) was separated from the suspension by centrifugation with the addition of flocculants and polymers. The input material as well as the silt, sand and gravel (2–8 mm) fractions were used for the column tests without further sieving or crushing (Figure 1). In addition, the aqueous solution (referred to as “washing water”) used for separation and cleaning of the solid fractions at the recycling plant was analyzed.

### 2.2. Experimental Setup

Prior to the column leaching tests, the gravimetric water content (*w*) of the solid fractions was determined by weighting and drying the wet material in an oven for 24 h at 105 °C. The dried material was further used to determine its volume and grain density using a gas pycnometer (micromeritrics/AccuPyc 1330). Quartz sand was used as a reference standard material (density: 2.65 g/cm^3^). The final values were set by measuring 10 times the same material until reaching a standard deviation of less than 0.005 g/cm^3^. In order to increase the permeability and to prevent mobilization of fine particles, the input material and silt fraction were mixed with clean quartz sand, as suggested elsewhere (e.g., [5]). From the input material, only particle fractions smaller than 32 mm were used (see Figure 1).

Column leaching tests were carried out according to the German standard DIN 19528 (2009) in a dark laboratory at a constant temperature of 20 °C [10]. The DIN 19528 has been validated for investigations on long-term leaching of salts and heavy metals from incineration bottom ash [9,21], comparisons with batch and lysimeters tests [3], and the effect of contact time in column percolation tests [9]. Furthermore, Lin et al. [5] recently proposed an optimization of the short column percolation tests (at L/S 2 eluates; DIN 19528,) by approving the use of sand admixtures in coarse grain fractions.

In total, 27 short column tests were performed for all fractions of the solid waste (i.e., RB, CDW1, CDW2 and CDW3), including 3 controls containing only a 3 cm layer of quartz sand. Short column eluates were collected until a L/S of 2 L/kg and analyzed for salts, metals, and organic substances such as BTEX, PCBs, herbicides and PAHs. In addition, 6 extensive column leaching tests were performed for sample CDW3 to examine the long-term leaching behavior of contaminants at L/S of 0.1, 0.3, 1, 2, 4 and 10 L/kg, including 1 control column. For the silt fraction, the earliest column eluate was collected at L/S 0.5 ratio. Glass columns with an inner diameter of 5 cm and a length of 30 cm were used for the sand and gravel fractions, whereas glass columns with an inner diameter of 7 cm were used for the input material and the silt fraction previously mixed with quartz sand. Before packing the samples into the columns, a 1 cm layer of quartz sand was placed at the bottom for better distribution of the water flow through the column inlet. A second quartz sand layer was placed at the top, at a filling height of about 28 cm, to prevent the release of fine particles. Additionally, glass wool was placed at the inlet and outlet openings. Teflon tubes were connected to the column inlets and the clean water reservoir consisting of a 50 L glass bottle containing Milli-Q water. The flow rate was set using a peristaltic pump (IPC 8, ISMATEC), and adjusted to allow a contact time of 5 h during the leaching tests. The initial flooding of the columns with clean water lasted approximately 2 h. Column eluates were collected in amber glass bottles at the corresponding L/S ratios and stored at a temperature of 20 °C until further analysis. Given that biodegradation and volatilization of organic compounds can occur, columns for PAHs were run in parallel—one for the analysis of ions and metals and the other for PAHs only. For PAH analysis, the collecting bottles previously contained 10 mL of cyclohexane (to avoid any biodegradation during sampling and storage), and an internal standard (10 μL, 5 perdeuterated PAHs according to DIN 38407–39 in toluene, each perdeuterated PAH 20 ng/μL). 

### 2.3. Analytics

#### 2.3.1. Turbidity, Electrical Conductivity, Ion Chromatography and DOC

All column eluates were analyzed for turbidity, electrical conductivity (EC, HACH LANGE), and pH (inoLab^®^ pH 7110, WTW) within the first 2 h after collection. After filtration at 0.45 μm, major ions were analyzed by ion chromatography (DIONEX, DX-120). Total Organic Carbon (TOC) and Dissolved Organic Carbon (DOC) were measured via a TOC analyzer (Elementar, Vario TOC). 

#### 2.3.2. Metals, Phenols, EOX, PCBs, PHCs, Cyanide and Herbicides

Solid concentrations of heavy metals, petroleum hydrocarbons (PHCs, C10-C40), polychlorinated biphenyls (PCB), phenols, extractable organic halides (EOX) and cyanide were analyzed at the Gewerbliches Institut für Umweltanalytik GmbH (Industrial Institute for Environmental Analysis, Teningen, Germany). PHCs, PCBs, phenols, EOX and cyanide concentrations were measured by gas chromatography−tandem mass spectrometry (GC-MS/MS, Agilent). 

Aqueous column eluates (aliquots of 20 mL) were filtered at 0.45 µm and acidified (HNO_3_) prior to the analysis of heavy metals via inductively coupled plasma mass spectrometry (ICP-MS, Agilent). The herbicides atrazine, simazine, bromacil, desethylatrazine, hexazinone, dimefuron, diuron, flumioxazin, thiazafluron and ethidimuron were measured in 20 mL aliquots of column eluates by liquid chromatography−tandem mass spectrometry (LC-MS/MS, Agilent). All substances were measured according to protocols described in [20].

#### 2.3.3. Polycyclic Aromatic Hydrocarbons (PAHs)

PAHs were measured in both solids and column eluates using GC-MS (Agilent/HP 5973). For solid concentrations, PAHs were extracted by Accelerated Solvent Extraction (ASE 300 DIONEX, Thermo Scientific), a technique that utilizes organic solvents at high temperature and pressures. Approximately 40 g of the solid samples were placed in the sample cell, along with 47 mm diameter filters on both ends of the extraction cell. Samples were extracted sequentially first with acetone and then with toluene (50 mL extracts) at a pressure of 100 bars and 100 °C [22]. Aqueous column eluates were extracted by liquid-liquid extraction. The bottles containing the column eluates along with 10 mL of cyclohexane (CH) and 10 μL of internal standard (10 μL, 5 perdeuterated PAHs according to DIN 38407–39, in toluene, each perdeuterated PAH 20 ng/μL) were horizontally shaken for 1 h (at 150 rpm), and subsequently filled with Milli-Q water until the solvent reached the bottleneck. The bottles were left overnight, and cyclohexane extracts were retrieved and treated with anhydrous sodium sulfate. All extracts were reduced to 200 μL by means of a nitrogen flow. 

## 3. Results and Discussion

### 3.1. Pollutant Screening in Solid Fractions

**Figure 2:**
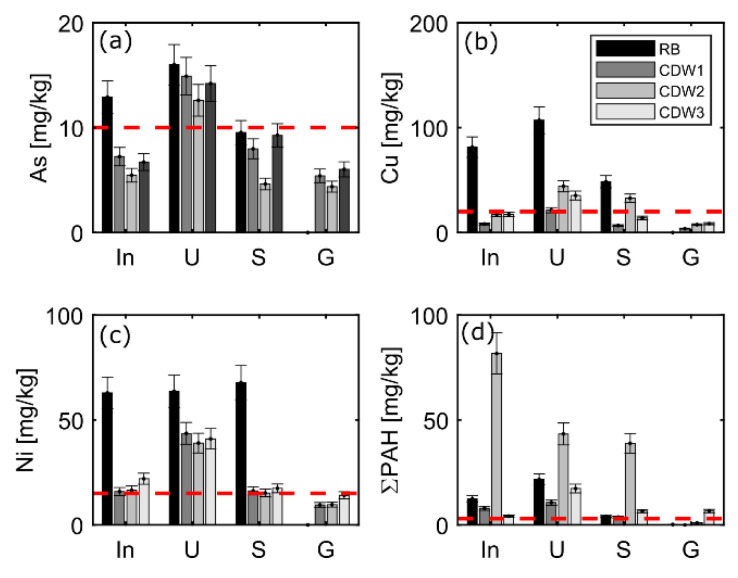
Concentrations of (**a**) As, (**b**) Cu, (**c**) Ni, and (**d**) the sum of the 16PAHs in solids: Input material (In), silt (U), sand (S) and gravel (G) of railway ballast (RB) and three sets of construction and demolition waste (CDW); input represents the material prior to separation into the different fractions (silt, sand and gravel), and the red dashed lines indicate precautionary values for sandy soils [19]. Error bars represent uncertainties in measurements.

Prior to the column tests, solids were analyzed for determination of initial concentrations and characterization of the materials according to precautionary values for soils [19]. Concentrations of PCBs were present in some solid fractions, but did not exceed precautionary values for soils (Appendix A, Table A1) [19]. Phenols, cyanide and EOX were not detected in the solid samples, except for the input material and the sand fraction of CDW2 with EOX concentrations of <0.5 µg/kg. PHCs (C10-C40) were present in concentrations below the limit value of material class BM-0* (<300 mg/kg; Table A1). For EOX and PHCs no precautionary values exist for soils [19]; therefore, limit values for material classes are used (Table A1). Metals exceeding precautionary values were detected in silt fractions of both RB and CDW materials, e.g., As (>10 mg/kg), Pb (>40 mg/kg), Cu (>20 mg/kg) and Zn (>60 mg/kg), while both, the silt and sand fractions exceeded the limit of solid concentrations for Cd (>0.4 mg/kg), Cr (>30 mg/kg) and Ni (>15 mg/kg) (Figure 2 and Table A1). Further metal solid concentrations are given in Table A1 for information.

Globally, the silt fraction was the most contaminated particularly with Cr and Cu in RB, and with the 16 PAHs in CDW samples (>3 mg/kg; Figure 2). The variability of solid concentrations in the different CDW samples is low for metals but high for PAHs. Moreover, RB shows different solid concentration patterns than CDW. The high variability of solid concentrations demonstrates that solid waste materials should preferably be examined as individual samples and according to grain size for intended use prior to recycling applications. While the washing process of the solid material into different grain size fractions should be considered as an important step for the separation of fractions suitable for recycling applications, the treatment is obviously not sufficient to clean up the materials to reach precautionary values for the sand and silt fractions. Only the gravel fraction reached concentrations below precautionary values (Figure 2), with the exception of PAHs in the gravel fraction of CDW3. The solid concentrations of PAHs in the sand and silt fraction of CDW 2 exceed the material value of RC-3 (20 mg/kg), and based on this, it cannot be reused and would have to be landfilled.

### 3.2. Contaminant Concentrations in Eluates of Short Leaching Tests and Washing Water

**Figure 3:**
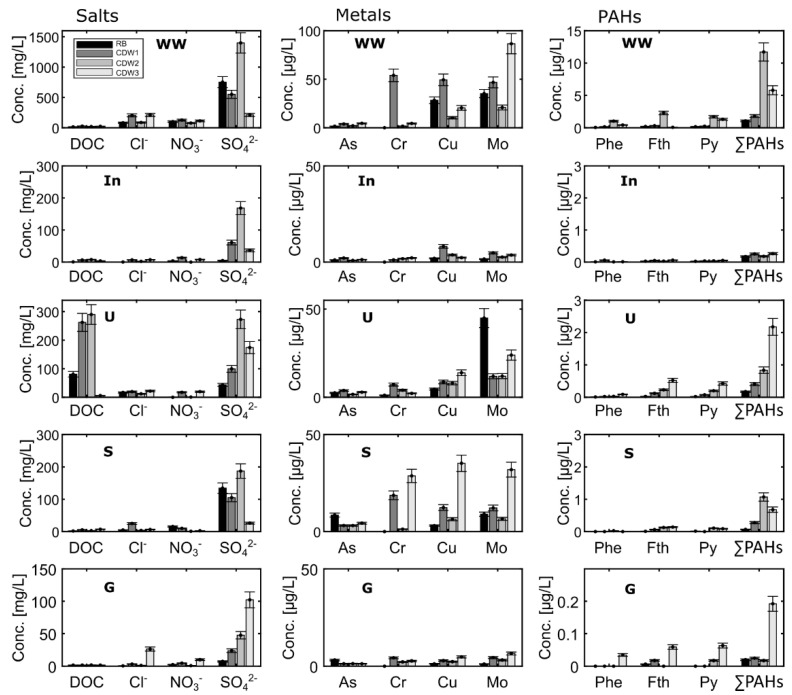
Concentrations of salts, metals and PAHs from short column tests at L/S 2 ratio of railway ballast (RB) and three sets of construction and demolition waste (CDW); WW represents concentrations in the washing water used for cleaning and separation of the input material (In) into the different size fractions silt (U), sand (S) and gravel (G). Error bars represent uncertainties in measurements.

Column eluates were examined at L/S 2 of the recycling materials RB, CDW1, CDW2 and CDW3 from 4 different solid fractions: input material (In), silt (U), sand (S) and gravel (G); column parameters are listed in Table 1. Further detailed concentrations are listed in the Supplementary Materials. The gravel fractions of both RB and CDW materials showed the lowest concentrations in the leachates, without exceeding GFS values, except for V (> 4 µg/L) and herbicides in RB (Appendix A, Table A2 and Supplementary Materials). Herbicides were only detected in RB (Table A2), where sand was the most contaminated fraction exceeding GFS values (>0.1 µg/L per herbicide). In general, leachates from CDW materials showed higher concentrations than those from RB, except for As and Mo (Figure 3). SO_4_^2−^ showed the highest concentrations up to 270 mg/L, followed by Cl^−^ and NO_3_^2−^. Furthermore, the highest eluate concentrations at L/S 2 were observed in silt fractions of CDW, followed by the input material, and sand and gravel fractions (Figure 3). Overall, the gravel fractions of RB and CDW materials proved to be the least contaminated, and thus suitable for a free re-use as recycling material in open technical applications.

Concentrations of salts, metals and PAHs were highest in column eluates of CDW3, particularly in U and S fractions (Figure 3). Notably, the concentrations of some salts and metals increased in U and S fractions compared to In, indicating a possible redistribution and accumulation of contaminants in the fine-grained fractions during the washing process. While concentrations in the solids of silt fractions were 2–3 times higher than in the sand fraction (see Figure 2), leaching at L/S 2 resulted in much lower concentrations in U compared to S in most cases, suggesting that metals bind stronger to finer particles (Figure 3) [23]. From the PAHs, phenanthrene, fluoranthene and pyrene showed the highest concentrations in column eluates of the different solid fractions (Figure 3). The sum of the 16 PAHs exceeded the GFS values (>0.2 µg/L) in all column eluates except those of the gravel fractions (Figure 3). The input material (In) shows concentrations that fall in between those observed for U, S and G fractions (Figure 3). The results obtained for CDW reflects the impact of material heterogeneity on contaminant leaching [24], which is highly variable in all cases.

In terms of usability of the solid waste material for different recycling purposes, the fine-solid fractions of RB and CDW are not suited for specific applications in technical constructions, which are sensitive with regard to groundwater protection (e.g., open applications with less than 1.5-m groundwater distance). These materials can be recycled only in isolated or semi-isolated applications with more than 1.5 m distance to the groundwater table and with suitable subsoil characteristics complying with the highest material classes (e.g., BM-F2 or BM-F3) [11,19]. As for the sand fraction of CDW, PAHs concentrations reached limit values for the best material class RC-1 of 4 µg/L. Overall, our results proved that the least contaminated fraction is the gravel (see Figure 2 and Figure 3), which is suitable for free re-use in all open applications in all technical constructions. Sand and silt fractions can be re-used as recycling material only in isolated or semi-isolated technical applications. Concerning contamination with PAHs, limited applications are possible only if the solid concentration limits for PAHs are met additionally, which is not always the case (PAHs exceed limits for recycling even after wet processing, e.g., CDW-2).

Solute concentrations were also measured in the washing water (WW, Figure 3) used during the separation of the solid materials into different grain-size fractions (i.e., silt, sand and gravel) at the recycling plant. The washing water showed concentrations of metals such as As, Cr, Cu and Mo up to 4.5 µg/L, 54 µg/L, 50 µg/L and 86 µg/L, respectively. The most dominant anions were Cl^−^ and SO_4_^2−^ with concentrations up to 212 mg/L and 550 mg/L, respectively. Aqueous concentrations of the sum of the 16 PAHs in WW reached up to 11.7 µg/L (Figure 3), particularly in CDW samples. Overall, the washing water (WW) showed concentrations exceeding the insignificance threshold values into groundwater (GFS values, [20]; see Table 2) and the limit values (methodological background values) for salts, and some of the metals and PAHs. These concentrations are in the range of material values of higher material classes as BM-F2 or RC-3 (Table 2) [19]. Therefore, the removal of contaminants during the washing process of solid waste material is essential to ensure adequate recycling fractions.

### 3.3. Comparison between Short and Extensive Column Tests: The Importance of Compliance Testing

**Figure 4:**
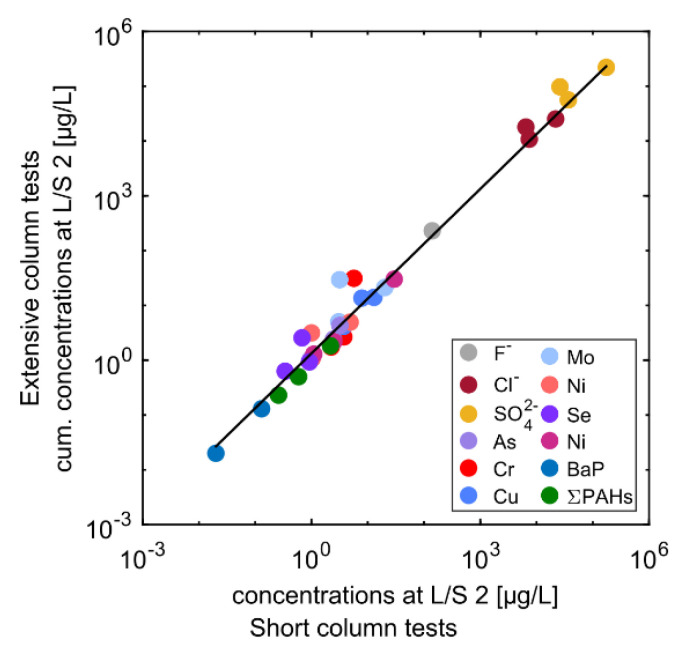
Comparison of short and extensive leaching test results for sample CDW3 (L/S 2); solid line represents the linear regression of the data (R^2^ = 0.92, slope = 1.33).

**Figure 5:**
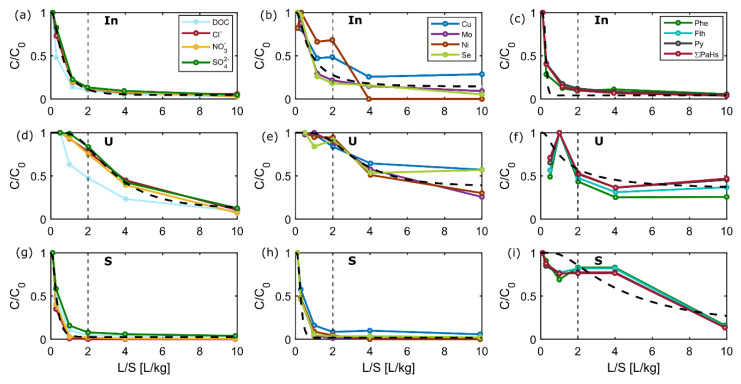
Normalized concentrations of selected groups of compounds and elements (“salts” left panel (**a**,**d**,**g**), “metals” middle panel (**b**,**e**,**h**), and PAHs right panel (**c**,**f**,**i**) vs. liquid-to-solid (L/S) ratio). Colored lines and symbols represent observations from extensive column test of different solid fractions of CDW 3: input material (In), silt (U), and sand (S); dashed lines represent the fitted results from the advection-dispersion model (with distribution coefficients *K_d_* ranging from 0.28–3.64 L/kg and *α/x* ratios from 0.07–0.50 for salts and metals).

**Figure 6:**
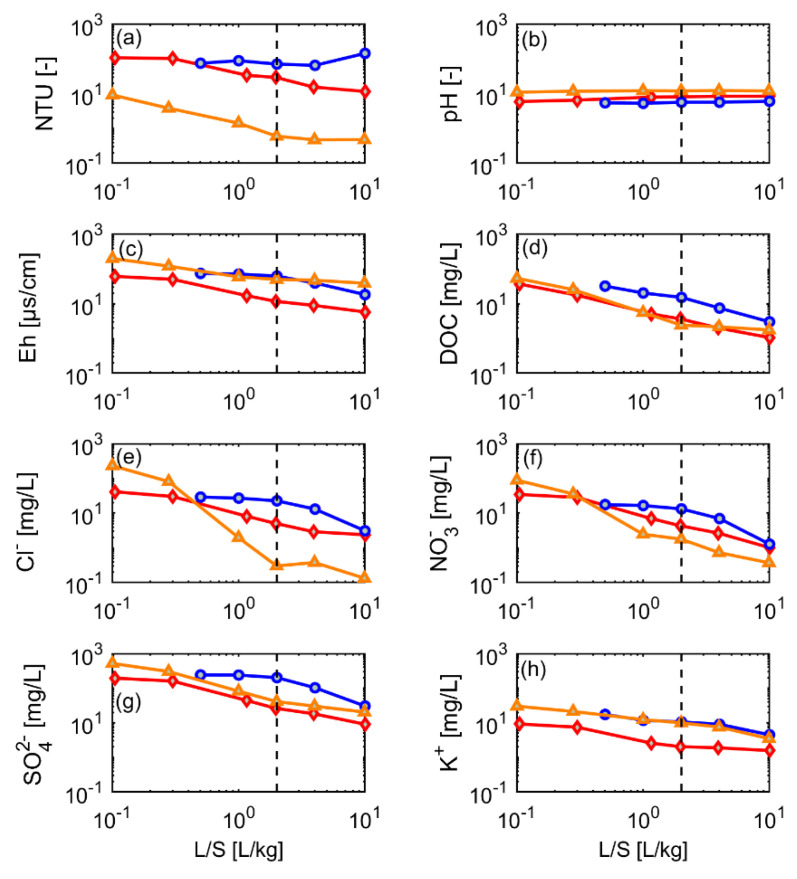
Leaching behavior of selected “salts” from different grain-size fractions of CDW material (CDW3) until a liquid-to-solid (L/S) ratio of 10 (dotted line L/S = 2); red diamonds: input material (mixture), blue circles: silt (<0.063 mm), orange triangles: sand (0–0.2 mm). (**a**) TSS, (**b**) pH, (**c**) Eh, (**d**) DOC, (**e**) Cl^−^, (**f**) NO_3_^−^, (**g**) SO_4_^2−^, and (**h**) K^+^.

**Figure 7:**
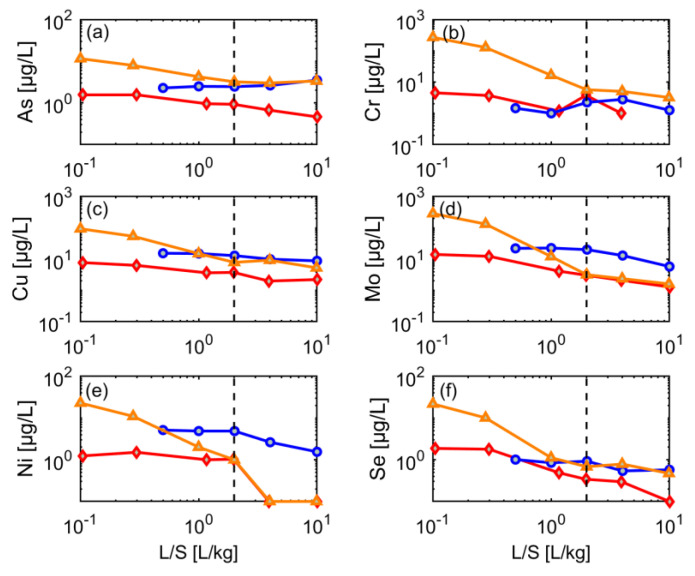
Leaching behavior of selected metals from different grain size fractions of CDW material (CDW3) until a liquid-to-solid (L/S) ratio of 10 (dotted line L/S = 2); red diamonds: input material (mixture), blue circles: silt (<0.063 mm), orange triangles: sand (0–0.2 mm). (**a**) As, (**b**) Cr, (**c**) Cu, (**d**) Mo, (**e**) Ni, and (**f**) Se.

**Figure 8:**
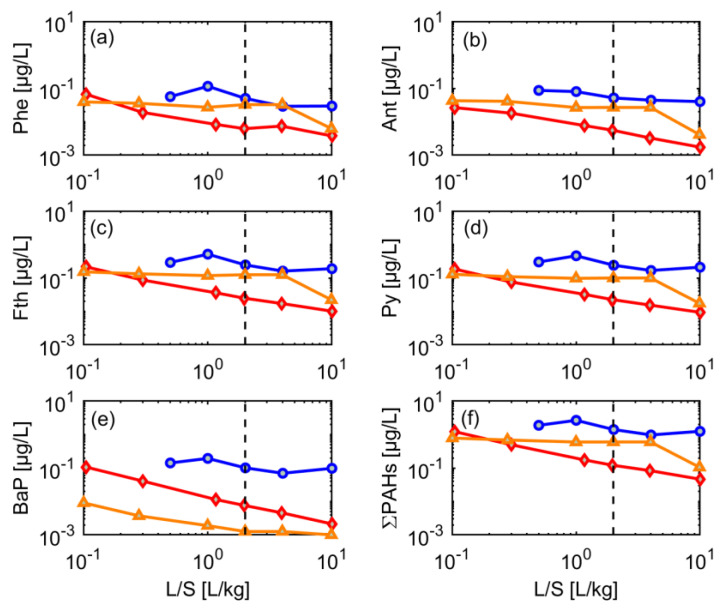
Leaching behavior of selected PAHs from different grain-size fractions of CDW material (CDW3) until a liquid-to-solid (L/S) ratio of 10 (dotted line L/S = 2); red diamonds: input material (mixture), blue circles: silt (<0.063 mm), orange triangles: sand (0–0.2 mm). (**a**) Phe, (**b**) Ant, (**c**) Fth, (**d**) Py, (**e**) BaP, and (**f**) the sum of 16PAHs.

Of the three sets of CDW (i.e., CDW1-CDW3), CDW3 material was selected to further examine the long-term leaching behavior of potential contaminants, as it proved to be the most contaminated solid material in L/S 2 eluates, particularly for the silt and sand fractions (Figure 3). The gravel fraction was not further examined as eluate concentrations in L/S 2 were lower than GFS values (see Figure 3 and Table 2). Figure 4 compares cumulative leaching in long-term to short-term tests at L/S 2 ratios. Figure 5 shows the grouping of salts, metals and PAHs in normalized leaching plots, and Figure 6, Figure 7 and Figure 8 show the dynamics of the long-term leaching behavior in log-log plots.

Long-term column tests were sampled from LS 0.1 (0.5 for silt) to L/S 10 (extensive tests) and analyzed for “salts”, metals and PAHs. Cumulative concentrations (*C_cum_*) were calculated from the cumulative mass released up to L/S 2 divided by the total volume of water at L/S 2. As expected, very good agreement was observed between aqueous concentrations from the short and extensive column tests at L/S 2 ratios proving that one-step short column tests are sufficient for compliance testing, and thus reduce testing time (Figure 4). Short-term column percolation tests are thus suitable for continuous internal (facility control) and continuous external quality control. Some variability was observed in the sand fraction, particularly for metals, which may be due to more complex solubility behavior relative to pH and redox conditions [21,25]. 

### 3.4. Typical Release Patterns of Groups of Substances and Fitting of the Advection-Dispersion Transport Model

The different mass-release pattern of salts, metals and PAHs observed in eluates of the extensive column tests demonstrated that substances can be grouped into rapid (“salts”), intermediate (“metals”) and slow leaching substances such as PAHs [3,4]. Figure 5 shows the similar leaching behavior of DOC, Cl^−^, NO_3_^−^ and SO_4_^2−^ (“salts”) based on normalized concentrations of the input material as well as silt and sand fractions. Metals such as Mo, Ni, Cu and Se may also be grouped, and showed a partially slower leaching than the “salts”. In general, slower leaching was observed in the silt fraction, probably due to smaller grain size and higher sorption capacity. PAHs showed decreasing concentrations only in the input material, while partly stable concentrations were observed for the silt and sand fractions. These similar leaching patterns were observed for Phe, Fth, Py and most of the other 16 PAHs (Figure 5).

As suggested earlier [3], a simple parsimonious transport model may be used to describe leaching in column tests, and to obtain average *K_d_* and longitudinal dispersivity (*α*) values by fitting to observed data (dashed line in Figure 5). A description of the model is provided in Appendix B. It should be noted that the fitting parameters *K_d_* and dispersivity (here *α/x* - *α* as a function of the length of the pack column *x*) lump together all processes that are not accounted for in the analytical solution, such as non-equilibrium sorption/desorption, non-linear sorption and/or slow desorption, which all lead to extended tailing and thus increased “dispersivity” (e.g., *α/x* > 0.12) [3]. While the approach worked reasonably well for “salts” and “metals”, PAHs did not follow the model well, probably also due to artifacts in measurements (Figure 5).

The estimated (fitted) average *K_d_* for salts and metals from eluates of the sand fraction were 0.28 to 0.32 L/kg, respectively, while eluates from the silt fraction resulted in the same *K_d_* values of 3.6 L/kg. For the input material, *K_d_* values of 0.7 and 1 L/kg were obtained for salts and metals, respectively. These results further support the high heterogeneity in the input material with a high fraction of silt leading to slower leaching (see also Figure 6 and Figure 7). Dispersivities fitted as *α/x* were 0.31–0.50 in the input material and thus larger than in silt (0.14–0.15) and sand (0.07–0.16) fractions, possibly also due to the pronounced heterogeneity of the input material. Overall, the results of the fitting model indicate that in most cases leaching initially occurs at or reasonably close to equilibrium, as indicated by Grathwohl and Susset (2009), in particular for the homogeneous material fractions. 

### 3.5. Dynamics of Contaminant Leaching

Figure 6, Figure 7 and Figure 8 illustrate the long-term dynamics of contaminant leaching in log-log plots. With the exception of TSS and pH, the “salts” (SO_4_^2−^, NO^3−^, NO^2−^, Cl^−^ and Na^+^) showed, initially, high concentrations (up to 400 mg/L, higher concentrations than in the short column tests but lower than in washing water), which decreased by 90% after L/S 2 in the sand fraction and input material (Figure 6). Silt showed the highest and most stable concentrations for PAHs and metals, which are attributed to high sorption capacity of the fine particles (Figure 7). In the sand fraction, a delayed decline in the metals was observed in some cases. The untreated input material showed mostly low and continuously decreasing concentrations, which likely results from the heterogeneity of the material largely composed by coarser fractions, which were least contaminated by PAHs and highly polluted fine materials (see Figure 2 and Figure 3). This probably leads to a superposition of solute leaching from different material classes and the typical power-law behavior observed for the input material in Figure 6, Figure 7 and Figure 8. Liu et al. (2021) [12] showed that heterogeneous mixtures of materials may result in very complex contaminant release characteristics in column leaching tests, especially if materials with different degrees of contamination are concerned. For example, a rapid initial decline in concentrations followed by concentration “tailing” maybe be explained by a heterogeneous material in which a small portion of less sorbing material (low *K_d_*, high *C_w,eq_*, low retardation) is mixed with a more strongly sorbing material (high *K_d_*, low *C_w,eq_*, high retardation) [12]. 

The leaching of metals typically varies from sample to sample and likely depends on several other parameters that change over time, such as pH, redox potential and ionic strength [3,15,26]. Here, initial metal concentrations were highest in the sand fraction (in contrast to the salts); Cr and Mo showed concentrations up to 286 µg/L and 269 µg/L (at L/S = 0.1 L/kg), respectively, followed by Cu (93 µg/L; Figure 7). Generally, metal concentrations decreased again by 90% at L/S 2 in the input material and sand fraction, including As, Ni and Se. 

The release pattern of PAHs also varied among the different grain-size fractions of CDW3. Initial concentrations of the 16 PAHs of the input material, silt and sand fractions were >0.2 µg/L (Figure 8). While concentrations of most PAHs were quite constant in the sand and silt fractions, indicating strong sorption; the input material showed a power-law behavior with continuously decreasing concentrations, as already observed for some of the metals and salts (see Figure 6 and Figure 7). The sudden drop in leachate concentrations at L/S 10 of the sand fraction is unclear and possibly reflects an artifact during the sampling procedure.

Generally, PAHs (and metals) may be associated to suspended particles or dissolved organic matter [27,28,29,30], but since turbidity and DOC remained well below 100 mg/L (see Figure 6), respectively, this would only affect strongly sorbing compounds with *K_d_* values larger than 10,000 L/kg [13]. DOC was always below 50 mg/L and continuously decreased to less than 10 mg/L (see Figure 6) in all fractions, which is not reflected in the rather stable concentrations of PAHs, e.g., in sand and silt fractions. Similarly, metals such as copper, which are known to form complexes with DOC showed fairly stable concentrations in the silt fraction, while DOC decreased rapidly. High TSS values were observed for silt and the input material, while the sand fraction showed very low and declining TSS values (see Figure 6). TSS in leachate of the silt fraction was quite stable and even showed an increase at L/S 10, while TSS of the input material dropped from 100 mg/L to 10 mg/L, which in principle could have affected leaching of high molecular weight PAHs (Fth, Pyr, BaP). Since all PAHs showed a similar leaching behavior, and concentrations in the sand fraction were higher than in the input material, particle facilitated transport seems not to play a major role (maybe with the exception of BaP, which, however, has the lowest concentrations and does not significantly contribute to the sum of PAHs). 

## 4. Conclusions

Wet processing after crushing of CDW and RB produces approximately 25 % of silt and sand, respectively, whereas the gravel fraction is usually around 50 %. Coarse-grained fractions (gravels) generally fulfilled legal standards for a free reuse in open technical applications (landscaping, etc.), while the sand fractions still showed concentrations which limit their reuse to specific technical applications. Fine-grain fractions (silt) are still contaminated and only allow limited re-use in (semi-) isolated applications, or require landfilling. This is also reflected in concentrations in solids and aqueous leachates up to L/S 2 (Figure 2 and Figure 3). 

Results from the short leaching tests showed to be comparable with the cumulative concentrations from the extensive column tests (up to L/S 2 L/kg; Figure 4). Thus, short leaching tests are suitable for compliance testing where concentrations can be compared to threshold values in order to select various material fractions for different recycling applications. Extensive column leaching tests showed, particularly for salts and some metals, a highly dynamic contaminant release with a decline to less than 10% of the initial concentration at *L/S* 2 for the sand fraction and input material. The silt fraction showed quite stable concentrations up to *L/S* 10, probably due to high sorption capacities for metals and PAHs. The leaching behavior of organic and inorganic substances from highly heterogeneous materials (i.e., “input material” of CDW 3) reflects their complex composition, making leaching patterns difficult to predict. As observed in earlier studies, a “typical” leaching behavior of highly soluble substances such as Cl^−^ and SO_4_^2−^, and metals such as Cu and Mo allows their grouping and can fit with simple transport models. Overall, short column leaching tests provide important information for decision making on the recycling of waste material. Future similar studies may help to optimize processing of mixed solid waste for higher recoveries of material fractions suitable for recycling.

## Figures and Tables

**Table 1:** Column parameters of short and extensive leaching tests of railway ballast (RB) and three sets of construction & demolition waste (CDW) materials. In: input material, U: silt fraction, S: sand fraction and G: gravel fraction.

Parameter	RB				CDW1				CDW2				CDW3				CDW3 Extensive Tests ^a^		
	In ^d^	U ^e^	S	G	In	U	S	G	In	U	S	G	In	U	S	G	In	U	S
Water content [%] ^b^	3.13	31.3	10.6	1.8	8.6	38.8	11.9	2.51	10.3	36.11	8.08	1.97	7.43	35.37	12.06	4.57	15	22	12.06
Dry sample [g]	1615	251	664	914	2417	182	676	882	1454	199	784	841	1326	193	873	840	927	172	839
Quartz sand [g]	1987	2225	-	-	2656	2208	-	-	2052	2192	-	-	2040	2208	-	-	1632	2507	-
Filling volume [cm^3^] ^b^	1193	1078	550	560	1116	1155	530	550	553	1116	550	530	550	1193	550	540	1155	1116	550
Porosity [%] ^b^	51	48	56	41	30	40	52	41	31	38	46	41	34	40	41	42	34	41	44
Flow velocity [mL/min]	1.96	1.03	0.79	0.85	1.11	1.52	0.84	0.41	0.54	1.5	0.83	0.74	0.62	1.55	0.74	0.72	1.30	1.48	0.80
TOC [mg C/g] ^c^	25.1	11.5	0.60	-	9.61	5.14	1.42	-	13.4	5.59	2.62	-	13.7	4.42	1.00	-	(see CDW3)		

^a^ Extensive leaching tests of material CDW3 only. ^b^ For the input material (In) and silt fraction (U), values represent the mixture of sample with dry quartz sand. ^c^ Total organic carbon. TOC was not measured in the gravel fraction due to expected insignificant organic carbon content. ^d^ Column parameters of the input material (In) are related to the columns performed for ions and metals analyses. ^e^ Column parameters of the silt fraction (U) are related to the columns performed for ions and metals analyses.

**Table 2:** “Insignificance threshold” concentrations (GFS) and material values of the examined organic and inorganic substances. GFS values are used to identify relevant substances in principle with regard to groundwater protection [20]. Material values are used for the classification of RB and CDW into material classes, which are linked with permissible applications in technical constructions, regulated in the upcoming German recycling degree [19].

Threshold and Material Values	F^−^	Cl^−^	SO_4_^2−^	As	Cr	Cu	Mo	Ni	Se	V	BaP	PAHs ^f^	PCBs ^g^	PHC ^h^	Phenol
	µg/L	mg/L	mg/L	µg/L	µg/L	µg/L	µg/L	µg/L	µg/L	µg/L	µg/L	µg/L	µg/L	µg/L	µg/L
GFS values ^a^	900	250	250	3.2	3.4	5.4	35	7	3	4	0.01	0.2	0.01	100	8
BM-F0* ^b^	-	-	250	12	15	30	55	30	-	30	-	0.3	0.02	150	12
BM-F1 ^c^	-	-	450	20	150	110	55	20	-	55	-	1.5	0.02	160	60
BM-F2 ^d^	-	-	450	85	290	170	55	20	-	450	-	3.8	0.02	160	60
RC-1 ^e^	-	-	600	-	150	110	-	-	-	120	-	4	-	-	-

^a^ Insignificance threshold values for groundwater protection (GFS values) [20]. ^b^ Material values with regard to technical constructions of soil materials BM-F0* [19]. ^c^ Material values with regard to technical constructions of soil materials BM-F1 [19]. ^d^ Material values with regard to technical constructions of soil materials BM-F2 [19]. ^e^ Material values with regard to technical constructions of RC-1 defined as the highest quality construction and demolition waste [19]. ^f^ 15 PAHs, excluding naphthalene and methylnaphthalene. ^g^ Sum of PCBs (PCB-28, -52, -101, -138, -153, -180) and PCB-118. ^h^ Limit concentrations of petroleum hydrocarbons ranging from C10 to C40.

## Data Availability

The data presented in this study are available in this article as Supplementary Information.

## Supplementary Materials

The following supporting information can be downloaded at: https://www.mdpi.com/article/10.3390/ma15030858/s1, Table S1: Chemical analyses of solids and leachates.

## Appendix A. Concentration Measurements

**Table A1:** Solid concentrations of railway ballast (RB) and three sets of construction and demolition waste (CDW) from different solid fractions. In: input material, U: silt fraction, S: sand fraction and G: gravel fraction. Values exceeding precautionary values in bold.

Samples	Sb	As	Ba	Pb	B	Cd	Cr	Co	Cu	Mo	Hg	Ni	Se	Tl	V	Zn	PHC ^a^	PCBs ^b^	BaP	16PAHs
		[mg/kg]							[mg/kg]					[mg/kg]					[mg/kg]	
RB-In	3.3	12.9	135	31	68.5	0.64	44.8	15.2	81.4	2.07	0.12	62.9	1.71	0.21	39.5	333	<25	0.003	0.92	12.50
RB-U	3.8	16	163	44.1	81.7	0.65	47.5	16	107	2.07	0.21	63.7	1.6	0.29	40.5	523	63	0.005	1.62	21.83
RB-S	2.9	9.3	139	16.8	49.5	0.35	38.8	13.9	48.5	1.6	0.06	67.8	1.76	0.1	36.4	125	86	0.009	0.26	4.08
CDW1-In	0.23	7.2	39.6	9.91	28	0.23	15.1	4.06	8.1	0.18	<0.05	15.9	2.09	0.11	21.5	34.9	<25	<0.002	0.65	7.91
CDW1-U	0.50	14.9	113	27.7	66.2	0.37	32.5	11.8	21.3	0.34	0.075	43.6	2.07	0.29	43.8	85.7	98	0.016	0.93	10.68
CDW1-S	0.20	7.9	36.9	13.8	22.6	0.12	13.6	3.82	6.6	0.35	0.05	16.2	<0.2	<0.1	17.2	32.6	139	0.004	0.27	3.717
CDW1-G	0.19	5.4	19.5	3.86	19.5	0.25	9.03	2.24	3.6	0.21	<0.05	9.5	1.84	<0.1	11.7	20.6	<25	<0.002	0.0	0.005
CDW2-In	0.44	5.4	90.5	15.8	32.6	0.16	15.2	4.79	17.3	0.33	0.07	16.5	0.31	0.15	19.8	45.4	121	0.011	6.03	81.89
CDW2-U	1.3	12.6	211	49.2	80.1	0.42	10.6	10.3	44.1	1.2	0.21	39	0.69	0.39	36.6	131	191	0.041	3.96	43.31
CDW2-S	0.61	4.6	66.1	15.6	22.8	0.25	13.8	4.31	32.7	0.50	<0.05	15.2	0.23	0.1	15.3	55.8	85	0.016	2.73	38.94
CDW2-G	0.65	4.3	53.4	13	18.1	0.17	9.2	2.4	7.4	0.21	<0.05	9.6	0.2	0.1	11.7	17	104	<0.002	0.08	0.94
CDW3-In	0.65	6.7	62	26.6	38.1	0.43	17.4	6.22	17.2	0.17	0.21	22	0.37	0.14	20.8	68	54	<0.002	0.33	4.18
CDW3-U	1.2	14.2	148	45.6	71	0.51	28.9	11.2	35.3	0.65	0.31	41	0.81	0.37	33.9	120	123	0.012	0.90	17.33
CDW3-S	0.42	9.2	74	26.1	27.2	0.36	13.8	5.13	13.9	0.32	0.05	17.4	0.45	0.11	18.7	35.9	93	<0.002	0.36	6.56
CDW3-G	0.25	6.0	38.5	7.37	24.3	0.20	10.3	3.6	8.4	0.19	<0.05	14.1	0.38	0.1	15.4	27.5	79	<0.002	0.51	6.62
Threshold ^c^	-	10	-	40	-	0.4	30	-	20	-	0.2	15	-	0.5	-	60	300 ^d^	0.05	0.3	3

^a^ Petroleum hydrocarbons of chain C10-C40. ^b^ Sum of PCBs (PCB28, PCB52, PCB101, PCB138, PCB153, PCB180). Precautionary values are given for PCB6 and PCB118 [19]. ^c^ Threshold concentration based on precautionary values in soils (BM-0 Sand) [19]. ^d^ Precautionary value of petroleum hydrocarbons (PHCs of C10-C40) based on limit value of “material class” BM-0* [19].

**Table A2:** Measured concentrations of herbicides in short leaching test of railway ballast (RB) on different solid fractions. In: input material, U: silt fraction, S: sand fraction and G: gravel fraction.

Samples	Atrazine	Simazine	Bromacil	Desethyl-atrazine	Diuron	Glyphosate	Ampa
	[µg/L]	[µg/L]	[µg/L]	[µg/L]	[µg/L]	[µg/L]	[µg/L]
RB-In	-	-	-	-	-	-	0.29
RB-U	0.05	0.04	-	0.045	0.046	-	-
RB-S	0.093	0.19	0.026	0.074	0.076	0.31	1.5
RB-G	0.027	0.042	-	-	0.032	0.15	0.43
RB-WW	0.062	0.1	0.029	0.079	0.023	-	0.29
GFS value ^a^	0.1	0.1	0.1	0.1	0.05	0.1	0.1
Threshold ^b^	0.2	0.2	0.2	-	0.1	0.2	2.5

^a^ Insignificant threshold values into groundwater [20]. ^b^ Threshold concentration for recycling railway ballast material (GS-0) [19].

## Appendix B. Long-Term Leaching Behavior Described by the Advection-Dispersion Equation

Column leaching tests represent the percolation of water through different types of solid materials. Initially, the column is saturated so that equilibrium conditions can be achieved rather rapidly (<5 h) [13]. The drop in concentrations is given by a change in non-equilibrium conditions leading to an extended tailing of low concentrations. The advection-dispersion model allows for the description of the movement of the front of clean water through the column as:

(A1) $$\frac{\partial C}{\partial t}=\frac{D}{R}\frac{\partial^{2}C}{\partial x^{2}}-\frac{v}{R}\frac{\partial C}{\partial x}$$

where *C* is the solute concentration, *D* is the longitudinal dispersion coefficient [m^2^ s^−1^], *v* is the average flow velocity [m s^−1^], *t* is time, *x* is the length of the column [m] and *R* denotes the retardation factor [-], defined as:

(A2) $$R=1+K_{d}\frac{\rho }{n}$$

where *K_d_* denotes the distribution coefficient [L/kg], defined as the ratio of concentrations in the solids to the aqueous concentrations (*C_s_*/*C_w_*), *ρ* is the dry bulk density [kg/L] and *n* is the porosity [-]. The advection–dispersion model assumes local equilibrium conditions, but previous studies demonstrated a reasonably well fit with the early leaching behavior [3,4]. This model is solved using the analytical solution for the movement of the front of clean water through the column [31] and expressed based on the dynamic liquid to solid ratio (L/S) as:

(A3) $$\frac{C}{C_{0}}=1-0.5\left[\mathrm{erfc}\left(\frac{K_{d}-LS}{2\sqrt{\frac{\alpha }{x}\left(\frac{n}{\rho }+K_{d}\right)LS}}\right)+\exp\left(\frac{x\left(1-\frac{1}{R}\right)}{\alpha }\right)\mathrm{erfc}\left(\frac{K_{d}-Ls}{2\sqrt{\frac{\alpha }{x}\left(\frac{n}{\rho }+K_{d}\right)LS}}\right)\left(1-\frac{C_{min}}{C_{0}}\right)\right]$$

where *C_min_* is the minimum concentrations usually detected at L/S 10 L/kg, and LS is the amount of water percolated through the column after *t* time relative to the dry weights of the solids in the column (= *v n t/x* *ρ*). The last term in brackets has been here added to fit the late data of the leaching tests, which show substantial tailing. Eq. B3 accounts for the initial displacement of low sorbing (high soluble) compounds during first flooding of the column. The model is fitted to measured data using MATLAB (v R2021b) and the function *lsqcurvefit*. From the fit, retardation factors (*R*) and distribution coefficients (*K_d_*) were calculated. In addition, the longitudinal dispersivity was fitted as a function of *x* (i.e., *α/x*). Maximum values of *K_d_* and *α* were set to 100 L/kg and 1 m, respectively.
